# Brain-derived neurotrophic factor protects against tau-related neurodegeneration of Alzheimer's disease

**DOI:** 10.1038/tp.2016.186

**Published:** 2016-10-04

**Authors:** S-S Jiao, L-L Shen, C Zhu, X-L Bu, Y-H Liu, C-H Liu, X-Q Yao, L-L Zhang, H-D Zhou, D G Walker, J Tan, J Götz, X-F Zhou, Y-J Wang

**Affiliations:** 1 Department of Neurology, Daping Hospital, Third Military Medical University, Chongqing, China; 2 Cadre Ward, Department of Neurology, Bethune International Peace Hospital, Shijiazhuang, China; 3 Laboratory of Neuroinflammation, Sun Health Research Institute, Sun City, AZ, USA; 4 Rashid Laboratory for Developmental Neurobiology, Silver Child Development Center, Morsani College of Medicine, University of South Florida, Tampa, FL, USA; 5 Clem Jones Centre for Ageing Dementia Research, Queensland Brain Institute, The University of Queensland, Brisbane, QLD, Australia; 6 School of Pharmacy and Medical Sciences and Sansom Institute, Division of Health Sciences, University of South Australia, Adelaide, SA, Australia

## Abstract

Reduced expression of brain-derived neurotrophic factor (BDNF) has a crucial role in the pathogenesis of Alzheimer's disease (AD), which is characterized with the formation of neuritic plaques consisting of amyloid-beta (Aβ) and neurofibrillary tangles composed of hyperphosphorylated tau protein. A growing body of evidence indicates a potential protective effect of BDNF against Aβ-induced neurotoxicity in AD mouse models. However, the direct therapeutic effect of BDNF supplement on tauopathy in AD remains to be established. Here, we found that the BDNF level was reduced in the serum and brain of AD patients and P301L transgenic mice (a mouse model of tauopathy). Intralateral ventricle injection of adeno-associated virus carrying the gene encoding human BDNF (AAV-BDNF) achieved stable expression of BDNF gene and restored the BDNF level in the brains of P301L mice. Restoration of the BDNF level attenuated behavioral deficits, prevented neuron loss, alleviated synaptic degeneration and reduced neuronal abnormality, but did not affect tau hyperphosphorylation level in the brains of P301L mice. Long-term expression of AAV-BDNF in the brain was well tolerated by the mice. These findings suggest that the gene delivery of BDNF is a promising treatment for tau-related neurodegeneration for AD and other neurodegenerative disorders with tauopathy.

## Introduction

Alzheimer's disease (AD) is the most common form of dementia, causing a progressive decline of cognitive functions. The pathological hallmarks of the disease are extracellular neurotic plaques consisting of amyloid-beta (Aβ) and intracellular neurofibrillary tangles consisting of aggregated hyperphosphorylated tau protein, as well as loss of synapses and neurons. Compelling evidence suggests that intracellular neurofibrillary tangles rather than neurotic plaques are more closely correlated with the cognitive decline and severity of AD.^1, 2^ In addition, concentrations of tau protein, detected in the cerebrospinal fluid of subjects at risk for AD, were increased 15 years before expected symptom onset.^3^ Given the evidence that the tau hyperphosphorylation is critical to induce neuronal death,^4, 5^ it has become widely recognized that tau-based therapeutic strategies may be not only effective for AD but also for other diseases with tauopathies. However, no effective therapeutics targeting tau-related neurodegeneration is available in clinical settings.

Brain-derived neurotrophic factor (BDNF), the most widely distributed neurotrophin in the central nervous system, has a pivotal role in synaptic plasticity and neuronal survival.^6^ Accumulating evidence indicates that BDNF polymorphisms and reduced BDNF expression in human brains are closely associated with the pathogenesis of AD. It is suggested that the C270T (a nucleotide substitution in a noncoding region) and Val66Met (a missense mutation at the codon 66) polymorphisms of the BDNF gene confer susceptibility to AD,^7, 8, 9, 10^ and AD subjects show reduced mRNA and protein levels of BDNF in the serum and brain as compared with healthy elderly controls.^11, 12, 13, 14^ Importantly, higher expression of BDNF slows down cognitive decline in the elderly, especially in the setting of advancing AD neuropathology, indicating that the brain BDNF level could be used as a novel marker for evaluating AD progression.^14, 15^ Accordingly, it is conceivable to increase BDNF levels by directly supplementing BDNF or indirectly stimulating BDNF expression as a potential disease-modifying approach for AD.^16^ Consistently, *in vitro* and *in vivo* experiments demonstrate that BDNF has neuroprotective effects against the cytotoxic effects and learning deficits induced by Aβ.^17, 18^ Similarly, BDNF gene delivery in Aβ precursor protein (APP)-transgenic mice improves learning and memory, and reserves synapse and cell loss caused by APP mutation.^19, 20^ In general, these lines of evidence suggest that direct application of BDNF exerts a protective effect against Aβ-related pathologies. However, no evidence is available that whether BDNF treatment can also ameliorate tauopathy of AD. In the present study, we investigated the therapeutic effects of BDNF on tauopathy in a P301L mouse model, using an adeno-associated virus (AAV)-mediated delivery method.

## Materials and methods

### Human sample collection and BDNF determination

Post-mortem human brain samples (parietal cortex) from histologically confirmed cases of AD (*n=*12) and healthy elderly controls (HE, *n=*12) were obtained from the Banner Sun Health Research Institute (Sun City, AZ, USA). Sex, age and education of the two groups are matched. Braak staging of AD group ranges from V to VI. The levels of BDNF in parietal cortex homogenates of AD patients and HE controls were quantified using western blot analysis (antibody from Abcam, Cambridge, MA, USA, ab72439). In addition, 44 probable AD patients and 54 age-matched HE controls were recruited from Daping Hospital of the Third Military Medical University. AD was diagnosed according to DSM-IV and the National Institute of Neurological and Communicative Disorders and Stroke (NINCDS-ADRDA) criteria. Serum samples were collected from all participants, and cerebrospinal fluid samples were collected from some participants. BDNF levels were quantified using human BDNF ELISA kits (Abbexa, Cambridge, UK) according to the manufacturer's instructions. The collection and subsequent biochemical analyses of serum and human brain tissues were approved by the Ethics Committee of Daping Hospital of the Third Military Medical University. Signed consent forms were obtained from all participants or their family members.

### Animals and sample collection

pR5 mice were imported from Australia and bred in the Daping Hospital animal house. This mouse model expresses P301L mutant tau transgene in neurons. The mice are characterized by age-related tau pathology including neuronal fibrillary tangle composed of hyperphosphorylated tau, memory impairment and mitochondrial dysfunction.^21^ All animals were maintained under standard conditions at 22 °C and a 12 h light/dark cycle with *ad libitum* food and water. Genotyping of mice was performed using PCR according to the supplier's instructions. Serum and brain homogenates from 18 P301L mice at different ages (4-, 8- and 12-month old, *n=*6 per age, half male and half female) and 18 age- and sex-matched wild-type (Wt) littermates (*n=*6 per age) were collected for the measurement of mouse BDNF using enzyme-linked immunosorbent assay (ELISA; Abbexa) and western blot assays. In addition, the levels of tau at different phosphorylation sites in brain homogenates were quantified using western blot analyses. A total of eight P301L transgenic mice with half male and half female were treated with intraventricular injections of AAV-BDNF at 3 months of age before the occurrence of significant neurofibrillary tangle formation, neuronal degeneration and cognitive impairment.^22^ As controls, eight P301L mice (four per sex) were treated with AAV-green fluorescent protein (AAV-GFP) at the same age. At 12 months of age, the treated P301L mice and additional eight P301L mice and eight Wt littermates (half male and half female) without any treatment were subjected to behavioral tests as controls. After behavioral testing, two mice from two AAV-treated groups were killed and 1 mm tissue blocks from the hippocampus were excised and fixed in 4% formaldehyde and 1% glutaraldehyde in 0.1 M PB (pH 7.4) for future transmission electron microscopy staining. The remaining mice were perfused transcardially with normal saline, and brains were harvested. The left hemisphere per animal was frozen for biochemical analysis. The right hemisphere for histological analysis was fixed in 4% paraformaldehyde (pH 7.4) for 24 h and incubated for 24 h in 30% sucrose for subsequent cryoprotection. Coronal sections of the brain were cut at 35 μm thickness with a cryosectioning microtome and stored at 4 °C in phosphate-buffered saline containing 0.1% sodium azide until use. All experiments were approved and documented by the Third Military Medical University Animal Welfare Committee.

### AAV-BDNF vector production and intraventricular injection

The vectors of AAV8-human mature BDNF (AAV-BDNF) and AAV8-enhanced GFP (AAV-GFP) were generated, produced and purified by Virovek (Hayward, CA, USA). Briefly, the target gene was cloned into Virovek's AAV production shuttle plasmid. Recombinant baculoviruses were generated and used to infect Sf9 cells to produce AAV vectors with Virovek's proprietary BAC-TO-AAV technology. The AAV vectors were purified by double CsCl ultracentrifugations and buffer-exchanged with PD-10 desalting columns. AAV titers were determined with quantitative real-time PCR and the purity was verified by SDS-PAGE, followed by SimplyBlue Safestain assay. P301L transgenic mice were injected with AAV-BDNF or AAV-GFP into the left lateral ventricle at 3 months of age. The injection coordinate point was taken from bregma following the stereotaxic coordinates: anteroposterior, −0.6 mm; lateral, 1.2 mm; and ventral, 2.2 mm. A total of 4 μl vector solution containing 2 × 10^9^ vector genomes of recombinant AAVs (AAV-BDNF or AAV-GFP, 0.5 × 10^9^ vg μl^−1^) were injected, which is equivalent to the dose used in clinical trials of AAV, in accordance with the Food and Drug Administration criteria for converting drug-equivalent dosages across species on the basis of body surface area: human equivalent dose in vg kg^−1^ = animal dose in vg kg^−1^ × (animal weight in kg per human weight in kg)^0.33^.

### Behavioral tests

Behavioral tests comprised the spontaneous alternation test, novel arm exploration test, object recognition test and Morris water maze test. In the spontaneous alternation test, mice were allowed to move freely through a Y-maze during a 5-min session. Alternation was defined as successive entries into the three arms on overlapping triplet sets. The percentage of alternation was calculated as the total number of alternation × 100/(total number of arm entries−2). In the Y-maze, the novel arm exploration test was also performed. First, one arm was blocked (defined as 'novel arm'), and mice were allowed to explore the other two arms ('home arm' and 'familiar arm') for 5 min. After a 2-h interval, mice were allowed to explore all three arms for 3 min freely. The number of novel arm entries and the time spent in the novel arm were recorded. The object recognition test was conducted in an open-field apparatus, and the test included habituation, familiarization and test phases. In the habituation phase, the mouse was placed in an empty open field and allowed to explore the apparatus for 3 min. After 24 h, the mouse was placed again in the apparatus and allowed to explore the two pre-placed identical objects for 3 min for familiarization. In the test phase, the two familiar objects (FOs) were replaced, one with the triplicate copy and the other by a novel object (NO). The mouse was placed in the apparatus for the third time to recognize the newly placed objects for 3 min. The exploration times for a FO and a NO were recorded, and a preference index (PI) was calculated as PI=NO time/(NO time+FO time). Before Morris water maze test, swimming ability of all mice were tested, and one mouse for Transgenic control group in treatment experiments was excluded because of disability of swimming. Morris water maze test consisted of three platform trials per day for five consecutive days, followed by a probe trial. Performance was video-recorded and analyzed with an image-analyzing software (ANY-maze, Stoelting, Wood Dale, IL, USA). In platform trials, the distance of path and escape latency were measured. In probe trials, the time spent in each quadrant and the number of annulus crossings were measured.

### Analysis of human BDNF expression

ELISA, western blot and immunohistochemistry assays were performed to examine the expression of human BDNF in the brains of P301L mice after intraventricular injection of AAV-BDNF. Fresh left hemisphere was weighed and homogenized in lysate buffer. The homogenates were centrifuged at 10  000 *g* for 10 min at 4 °C, and the resultant supernatant was collected and subjected to ELISA assays according to the manufacturer's protocols (human BDNF ELISA kit, Abbexa). In addition, the levels of total BDNF including endogenous mouse BDNF and human BDNF in brain homogenates were also detected by western blot analysis using anti-BDNF antibody (Abcam, ab72439, detecting both mouse and human BDNF). Coronal sections from the right hemisphere were immunostained for microglia (anti-CD45 antibody, Millipore, Bedford, MA, USA), astrocyte (anti-glial fibrillary acidic protein antibody, Millipore), neurons (anti-NeuN antibody, Abcam), GFP (anti-GFP antibody, Abcam) and human BDNF (anti-human BDNF antibody, R&D systems, Minneapolis, MN, USA) to identify localization and levels of human BDNF expression in P301L mouse brains after AAV-BDNF injection.

### Immunostaining

A series of five equally spaced tissue sections (∼1.3 mm apart) spanning the entire brain were subjected to immunostaining using the free-floating method as described previously.^23^ Primary antibodies to NeuN (Abcam), choline acetyltransferase (ChAT; Abcam), pS396-Tau (Signalway, Pearland, TX, USA), postsynaptic density protein 95 (PSD-95; Millipore) and vesicle-associated membrane protein 1 (VAMP-1; Epitomics, Burlingame, CA, USA) antibodies were used in a 1:50–1:200 dilution. After incubation with primary antibodies for 24 h, the sections were incubated with secondary antibodies and developed with 3,3'-Diaminobenzidine or Alexa Fluor fluorescent dyes. Images of immunostaining were collected and quantified using ImageJ. The number of neurons (NeuN^+^) in the same part of CA1 region in a series of five slices with an equal space across the hippocampus was counted, and the number of ChAT-positive neurons per visual field in the basal forebrain, including the medial septum, intermediate part of the lateral septum and vertical limb of the diagonal band, was counted (five slices with an equal space from the basal forebrain per animal were selected, and the neuron number of same regions per slice was recorded), following methods described previously.^24, 25^

### ELISA assays of pT181 and pT231

Brain homogenates were extracted successively with Tris buffer solution (TBS) and 2% sodium dodecyl sulfonate (SDS) buffer in the presence of phosphatase inhibitors. Levels of human pT181 and pT231 in the TBS and SDS fractions were determined according to the supplier's instructions (Novex, KH00631 for pT181, KHB8051 for pT231).

### Western blotting

Frozen fresh brain samples were homogenized in lysis buffer in the presence of protease inhibitors or phosphatase inhibitors. Samples were separated by SDS-PAGE (4–10% acrylamide) and transferred to nitrocellulose membranes. The blots were probed with the following antibodies: pS396-tau (Signalway), pS202/T205-tau (Signalway), pS404-tau (Signalway), glycogen synthase kinase 3 beta (GSK3β Abcam), pS9-GSK3β (Abcam), protein phosphatase 2A (PP2A, Millipore), PP2A-Y307 (Millipore), synaptosomal-associated protein 25 (SNAP-25, Millipore), synapsin I (SynI, Millipore), VAMP-1 (Epitomics), PSD-95 (Millipore), postsynaptic density protein 93 (PSD-93, Abcam), doublecortin (Abcam), nestin (Millipore), neuron-specific enolase (Abcam), neurofilament-200 (Abcam), ChAT (Abcam), NeuN (Abcam), microtubule-associated protein 2 (Millipore) and β-actin (Sigma-Aldrich, St Louis, MO, USA). The membranes were incubated with IRDye 800CW secondary antibodies (Li-COR) and scanned using the Odyssey fluorescent scanner. The band density was normalized to β-actin or NeuN when analyzing, as appropriate.

### Transmission electron microscopy staining

After fixation in 4% formaldehyde and 1% glutaraldehyde overnight, hippocampal tissue blocks were successively subjected to post-fixation, embedding and sectioning. Images of pyramidal neuron were collected using a Joel 1200 EX transmission electron microscopy.

### Statistical analysis

Unless otherwise stated, the results are presented as mean±s.e.m. The data were first assessed for normal distribution using the one-sample Kolmogorov–Smirnov test. Statistical comparisons between two groups were tested using Student's *t*-test, or Mann–Whitney *U*-test, as applicable. Comparisons among groups were determined using the one-way analysis of variance, or Kruskal–Wallis test, as appropriate. Categorical variables were compared by *χ*^2^-test, or Fisher exact test, as applicable. *P-*values<0.05 were considered significant.

## Results

### BDNF levels are reduced in the blood and brain of AD patients and P301L transgenic mice

**Figure 1:**
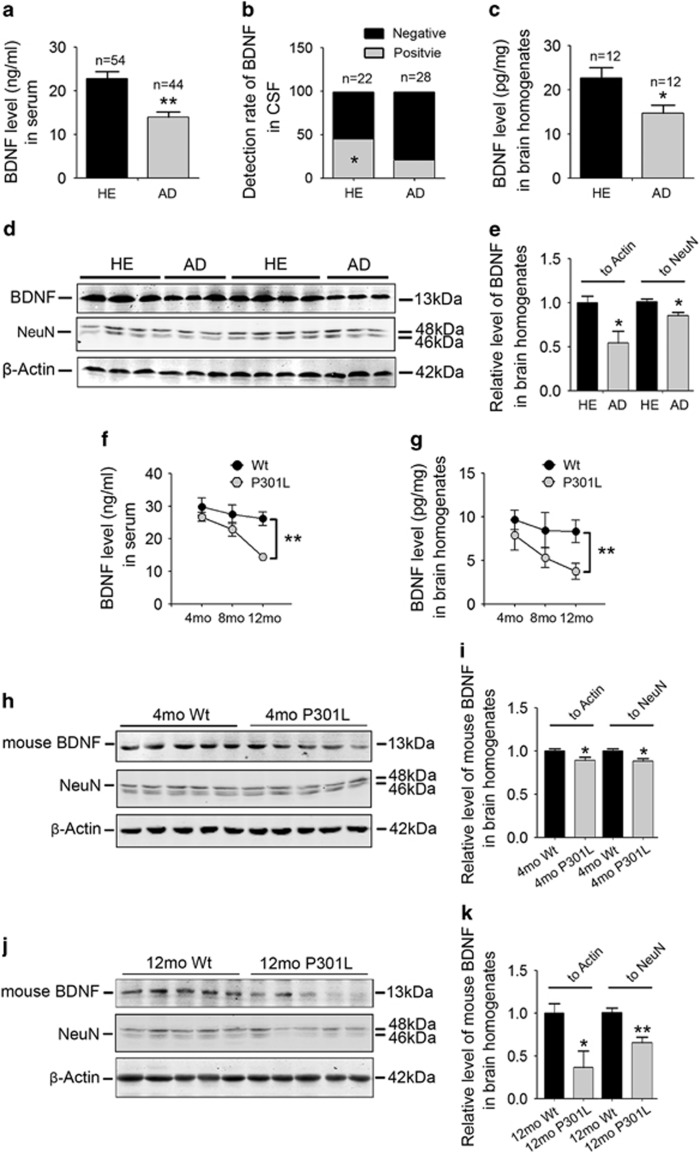
Brain-derived neurotrophic factor (BDNF) protein levels in serum and brain are reduced in Alzheimer's disease (AD) patients and P301L transgenic mice. (**a)** Comparison of BDNF levels in serum between AD patients and age- and sex-matched healthy elderly controls (HE; mean±s.e.m., Student's *t*-test, **P<0.01). (**b**) Detection rate of BDNF in cerebrospinal fluid (CSF) by enzyme-linked immunosorbent assay (ELISA; %, *χ*^2^-test, *P<0.05). (**c**) Comparison of BDNF levels in serum between AD patients and HE controls (detected by ELISA) (mean±s.e.m., Student's *t*-test, *P<0.05). (**d**, **e**) Representative western blot images (**d**) and quantification (**e**) of BNDF expression in brain homogenates from pathologically confirmed AD patients and age-matched HE (mean±s.e.m., *n=*12, Student's *t*-test, *P<0.05). (**f**, **g**) BDNF levels in serum (**f**) and brain homogenates (**g**) of P301L tau transgenic pR5 mice and their wild-type (Wt) littermate controls at different ages (mean±s.e.m., *n=*6 per age and per type, two-way analysis of variance (ANOVA), Tukey's test, **P<0.01). (**h**, **i**) Representative western blot images (**h**) and quantification (**i**) of mouse BDNF in brain homogenates of 4-month-old mice (mean±s.e.m., *n=*6 per age and per type, Student's *t-*test, *P<0.05). 4mo P301L denotes 4-month old P301L transgenic mouse; 4mo Wt denotes 4-month-old Wt littermates. (**j**, **k**) Representative western blot images (**j**) and quantification (**k**) of mouse BDNF in brain homogenates of 12-month-old mice (mean±s.e.m., *n=*6 per age and per type, Student's *t-*test, *P<0.05, **P<0.01).

Serum BDNF was significantly reduced in AD patients compared with age- and sex-matched HE controls (Figure 1a, source data of participants shown in Supplementary Table), and the detection rate of cerebrospinal fluid BDNF was lower in the AD group than in the HE group (Figure 1b). BDNF levels in parietal cortex homogenates of pathologically confirmed AD patients (Braak staging V–VI) were decreased by ~50% compared with age- and sex-matched HE controls, as quantified using ELISA (Figure 1c) and western blot analyses (Figure 1d, e). To investigate whether the P301L mouse had similar BDNF changes, we conducted ELISA and western blot assays to determine BDNF concentrations of serum samples and brain tissue homogenates of the mice at different ages. From 4 months of age, P301L mice began to show BDNF reduction in both blood and brains compared with age- and sex-matched Wt littermate controls, and the extent of BDNF reduction became more obvious with aging (Figure 1f, g). As expected, at 12 month of age, P301L mice had higher phosphorylated tau levels in the brains than their Wt littermates, as detected by western blots (Supplementary Figure 1). Next, we further determined whether the BDNF reduction was independent of neuron loss, the characteristic of tauopathy. Using NeuN, a widely used neuron marker, as an internal control, we quantified relative levels of BNDF in the brain homogenates of P301L mice and Wt controls by western blotting. We found that as early as at 4 month of age, when neuron loss was not detectable in the brains of P301L mice,^22^ the relative brain BDNF level has become lower in P301L mice than in Wt controls (with 15% reduction; Figure 1h, i). At 12 months of age, the reduction of relative brain BDNF level in P301L mice was more significant (Figure 1j, k). These findings suggest that BDNF deficiency in the brains of AD patients or P301L mouse models is not secondary to neuron loss.

### Restoration of brain BDNF levels in P301L mice by delivery of recombinant human BDNF gene using the AAV8 vector

**Figure 2:**
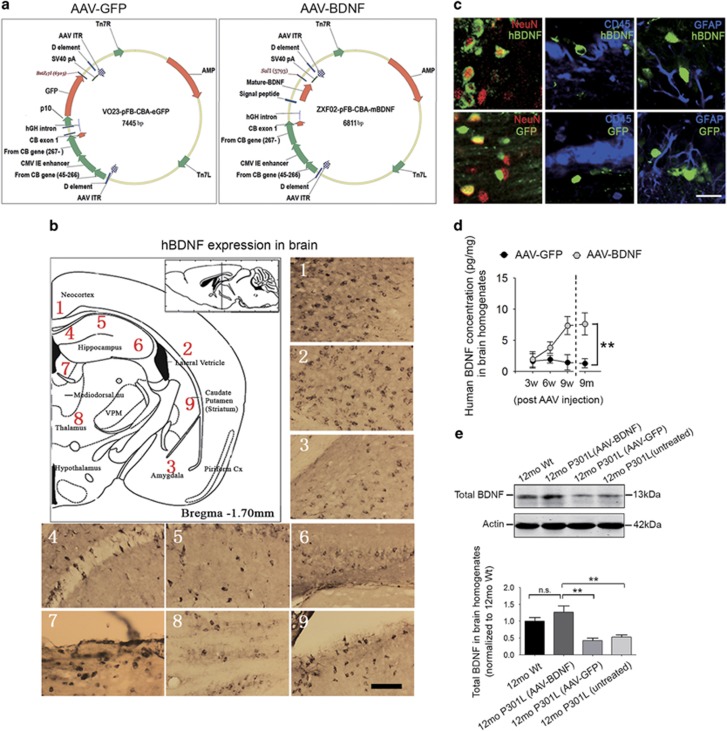
Wide and stable expression of human brain-derived neurotrophic factor (BDNF) after intraventricular injection of adeno-associated virus carrying BDNF (AAV-BDNF). (**a**) Schematics of the construction and characterization of AAV-green fluorescent protein (AAV-GFP; left panel) and AAV-BDNF (right panel) vectors. (**b**, **c**) In order to exclude endogenous mouse BDNF, we applied a specific anti-human BDNF antibody to detect the expression of human BDNF within P301L mouse brain. Human BDNF expression is detectable in multiple brain regions (**b**), and GFP or human BDNF was selectively expressed in neurons but not in microglia (CD45-positive) or astrocytes (glial fibrillary acidic protein (GFAP)-positive) (**c**). Scale bar=80μm (**b**) or 40μm (**c**). (**d**) Comparison of human BDNF levels in brain homogenates of P301L mice treated with AAV-BDNF or AAV-GFP at 3 weeks (3w), 6 weeks (6w), 9 weeks (9w) and 9 months (9 m) after AAV injection initiated at 3 months of age (*n=*4 per group for 3w, 6w and 9w; *n=*6 for 9 m group; mean±s.e.m.; two-way analysis of variance (ANOVA), Tukey's test, **P<0.01). The concentrations of human BDNF were determined using specific human BDNF enzyme-linked immunosorbent assay (ELISA) kits. (**e**) Western analysis of total BDNF expression (including endogenous mouse BDNF and exogenous human BDNF) in brain homogenates (*n=*5 per group; mean±s.e.m., one-way ANOVA, Tukey's test, **P<0.05; n.s. denotes no significant difference). 12mo P301L (AAV-BDNF) and 12mo P301L (AAV-GFP) denote 12-month-old P301L mice treated with AAV-BDNF and AAV-GFP, respectively; 12mo P301L (untreated) denotes untreated 12-month-old P301L mice; 12mo Wt denotes age-matched wild-type littermates.

The AAV8 vector was used to deliver the human mature BDNF gene into the brain of P301L mice at 3 months of age, before the occurrence of significant neurofibrillary tangle formation, neuronal degeneration and cognitive decline. AAV-GFP at the same dose was used as a control. The schematic of AAV vector construction is shown in Figure 2a. Using specific antibodies, we found that GFP and human BDNF were widely expressed in different brain regions (Figure 2b and Supplementary Figure 2), indicating the penetrating ability of AAVs from the ventricles into the brain parenchyma, as well as the potent transporting capacity of AAVs to distal projection sites because of its neurotropic characteristic. Both GFP and human BDNF were expressed selectively in neurons, but not in CD45- or glial fibrillary acidic protein-positive glial cells (Figure 2c). Human BDNF expression in brains could be detected at 3 weeks after AAV injection (Figure 2d), and it remained stable from 9 weeks to 9 months post injection (Figure 2d). Total BDNF levels in the brains after stable expression of human BDNF was comparable to the physiological concentrations of age- and sex-matched Wt littermates, and significantly higher than that of AAV-GFP-treated P301L mice and untreated P301L mice (Figure 2e).

### Restoration of the brain BDNF level rescues the behavioral deficits that characterizes P301L mice

**Figure 3:**
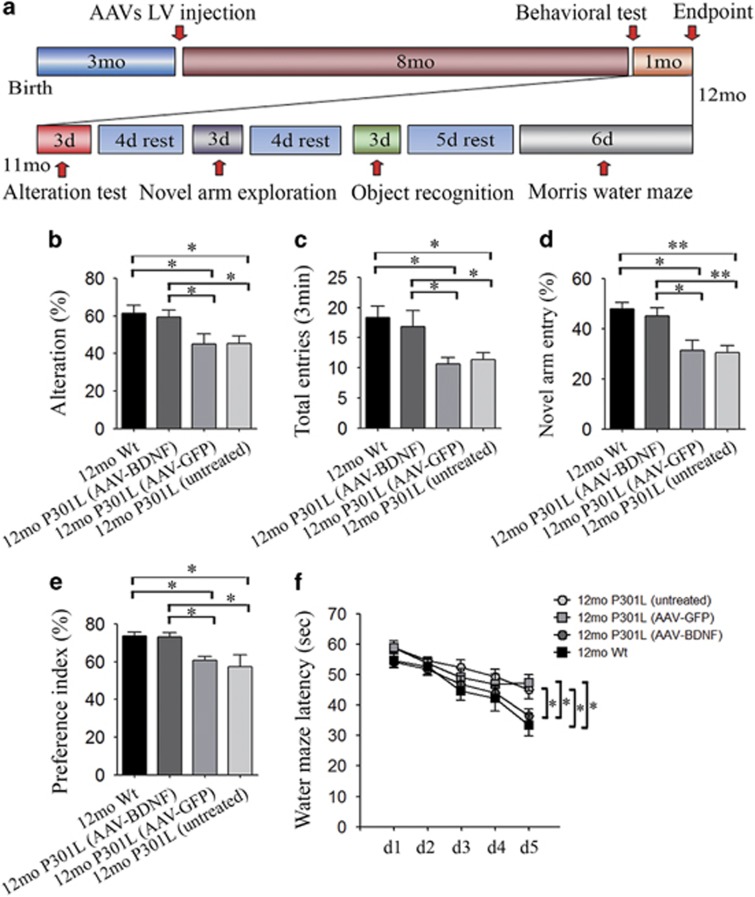
Delivery of adeno-associated virus carrying brain-derived neurotrophic factor (AAV-BDNF) rescues the behavioral impairment of P301L mice. (**a**) Time schedule of behavioral tests. (**b**, **c**) The alternation percentage (**b**) and total number of entries (**c**) in the spontaneous alternation test (mean±s.e.m.; *n=*8 per group, one-way analysis of variance (ANOVA), Tukey's test, *P<0.05). (**d**) Comparison of the percentages of novel arm entries among different groups (mean±s.e.m.; *n=*8 per group, one-way ANOVA, Tukey's test, *P<0.05, **P<0.01). (**e**) Comparison of the preference index in the novel object recognition test among different groups (mean±s.e.m.; *n=*8 per group, one-way ANOVA, Tukey's test, *P<0.05). (**f**) Escape latency (seconds) during platform trials in Morris water maze (mean±s.e.m., *n=*8 per group, two-way ANOVA, Tukey's test, *P<0.05). GFP, green fluorescent protein; Wt, wild-type.

Nine months after AAV delivery, a series of behavioral tests were conducted (scheme in Figure 3a). As expected,^22, 26^ compared with Wt littermates, untreated P301L mice showed significant cognitive decline in multiple cognition domains observed through Morris water maze, Y-maze and NO recognition tests (Figure 3b–f and Supplementary Figure 3c). AAV-GFP-treated P301L mice had similar behavioral performances to untreated P301L mice (Figure 3b–f and Supplementary Figure 3c), indicating that the injection of AAV-GFP and expression of GFP had no effect on the cognition of P301L mice. Compared with intact or AAV-GFP-treated P301L mice, the P301L mice treated with AAV-BDNF performed better in the Y-maze, as reflected by a significant increase in the alternation percentage (Figure 3b) and total entry number (Figure 3c) in the spontaneous alternation test, and a higher percentage of novel arm entry (Figure 3d). Moreover, the PI for the NO (procedures shown in Supplementary Figure 3a) of the AAV-BDNF group was significantly higher than that of the AAV-GFP and untreated P301L groups (Figure 3e). In the Morris water maze test, AAV-BDNF-treated P301L mice showed shorter escape latency (Figure 3f) and more annulus crossing (Supplementary Figure 3c). There was no difference in swim speed among groups (Supplementary Figure 3b). These data indicate that the AAV-BDNF treatment significantly attenuates the behavioral deficits of P301L mice.

### Restoration of the brain BDNF level prevents neuronal loss, synaptic degeneration and neurogenesis impairment in P301L mice

**Figure 4:**
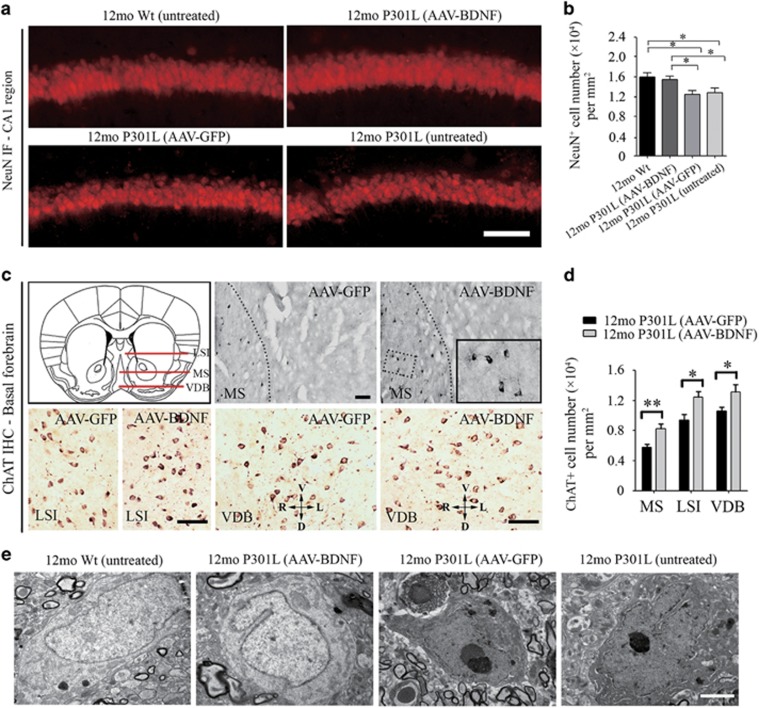
Neuroprotective effects of adeno-associated virus carrying brain-derived neurotrophic factor (AAV-BDNF) on neurons. (**a**, **b**) Representative images (**a**) and quantitative statistics (**b**) of neurons in the CA1 region of the hippocampus of P301L mice (untreated, AAV-BDNF treated and AAV-green fluorescent protein (AAV-GFP) treated) and wild-type (Wt) littermates, using NeuN immunofluorescence (IF) assays (six mice per group; mean±s.e.m., one-way analysis of variance (ANOVA), Tukey's test, *P<0.05, **P<0.01). Scale bar=25μm. (**c**) Schematic of basal forebrain in the coronal plane (upper-left panel) and representative images of choline acetyltransferase (ChAT)-positive cholinergic neurons in the medial septum (MS), intermediate part of the lateral septum (LSI) and vertical limb of the diagonal band (VDB) in AAV-BDNF and AAV-GFP groups. The inset shows the representative morphology of cholinergic neurons in MS at a higher magnification. V, ventral; D, dorsal; L, left; R, right. Scale bar=25μm. (**d**) Comparison of numbers of cholinergic neurons in MS, LSI and VDB between the two groups (six mice per group; mean±s.e.m., Student's *t*-test, *P<0.05, **P<0.01). (**e**) Representative electron micrographs of pyramidal neurons from the CA1 region of the hippocampus in the four groups. It is worth noticing that in 12mo P301L (AAV-GFP) group and untreated P301L group, a number of pyramidal cells shows structural changes typical of apoptosis, including an enlarged perinuclear space, chromatic agglutination and karyopyknosis, whereas apoptosis-like cells were rarely found in 12mo P301L (AAV-BDNF) group, and pyramidal cells in this group usually displayed with uniform chromatin density and a large nucleus, which was similar to 12mo Wt group. Scale bar=2μm.

**Figure 5:**
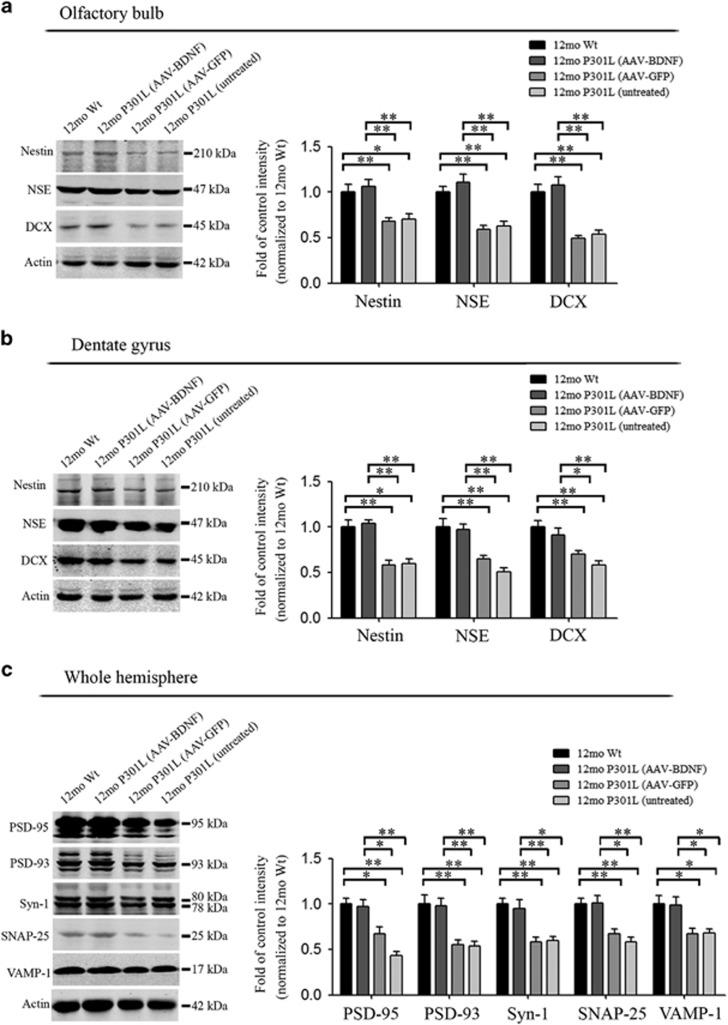
Supportive effects of adeno-associated virus carrying brain-derived neurotrophic factor (AAV-BDNF) on neurogenesis and synapse. (**a**, **b**) Representative western blot images and quantitative analyses of the expressions of nestin, neuron-specific enolase (NSE) and doublecortin (DCX) in olfactory bulb (**a**) and dentate gyrus (**b**) of the four groups (*n=*6 per group, mean±s.e.m., one-way analysis of variance (ANOVA), Tukey's test, *P<0.05, **P<0.01). (**c**) Representative western blot images and quantitative analyses for PSD-95, PSD-93, Syn-1, SNAP-25 and VAMP-1 expression in brain homogenates of the four groups (*n=*6 per group, mean±s.e.m., one-way ANOVA, Tukey's test, *P<0.05, **P<0.01).

Neuronal loss and synaptic degeneration, as well as impaired neurogenesis, are the pathological bases accounting for behavioral deficits. To investigate the mechanism underlying the improvement of behavioral performances following AAV-BDNF treatment, we tested the effects of BDNF supplementation on these pathologies in P301L mice. Consistent with previous studies,^22^ 12-month-old P301L mice had remarkable neuronal loss in the CA1 region of hippocampus (Figure 4a, b), and significant synaptic degeneration and neuronal impairment as compared with age-matched Wt mice (Figure 5). We also found that the expression of multiple neuronal structure biomarkers, including microtubule-associated protein 2, neurofilament-200 and NeuN, in the brain of 12-month-old P301L mice was significantly lower than those in their Wt littermates (Supplementary Figure 4). AAV-GFP treatment has no effect on these markers in the brains of P301L mice, as reflected by comparable levels of NeuN, microtubule-associated protein 2, neurofilament-200, ChAT, Nestin, NES, doublecortin, PSD-93, PSD-95, Syn-1, SNAP-25 and VAMP-1 in AAV-GFP-treated P301L mice to untreated P301L mice (Figures 4 and 5 and Supplementary Figure 4). However, compared with the AAV-GFP group, numbers of total neurons and cholinergic neurons were significantly increased in the AAV-BDNF group, as reflected by an increase in the number of NeuN-positive neurons in the CA1 region of hippocampus (Figure 4a, b) and ChAT-positive neurons in the basal forebain, respectively (Figure 4c, d). It was noticed that a subset of pyramidal neurons in the hippocampus of control and AAV-GFP-treated P301L mice typically displayed some structural changes indicative of apoptosis, including an enlarged perinuclear space, chromatin condensation and karyopyknosis, whereas apoptosis-like neurons were rarely found in the AAV-BDNF group (Figure 4e). It is consistent with the findings in western blot analysis for neuronal structure biomarkers (Supplementary Figure 4). Importantly, using western blot assays, we found that in dentate gyrus and olfactory bulb, where neurogenesis occurs actively, the expression of multiple new neural biomarkers, including Nestin, neuron-specific enolase and doublecortin in the AAV-BDNF group, was significantly higher than those in the AAV-GFP group (Figure 5a, b). In addition, following AAV-BDNF injection, several synaptic proteins, such as PSD-95, PSD-93, syn-1, SNAP-25 and VAMP-1, were upregulated in the brains of P301L mice (Figure 5c and Supplementary Figure 5), suggesting an protective effect of BNDF supplementation against synaptic degeneration.

### Delivery of recombinant human BDNF gene does not affect tau hyperphosphorylation

**Figure 6:**
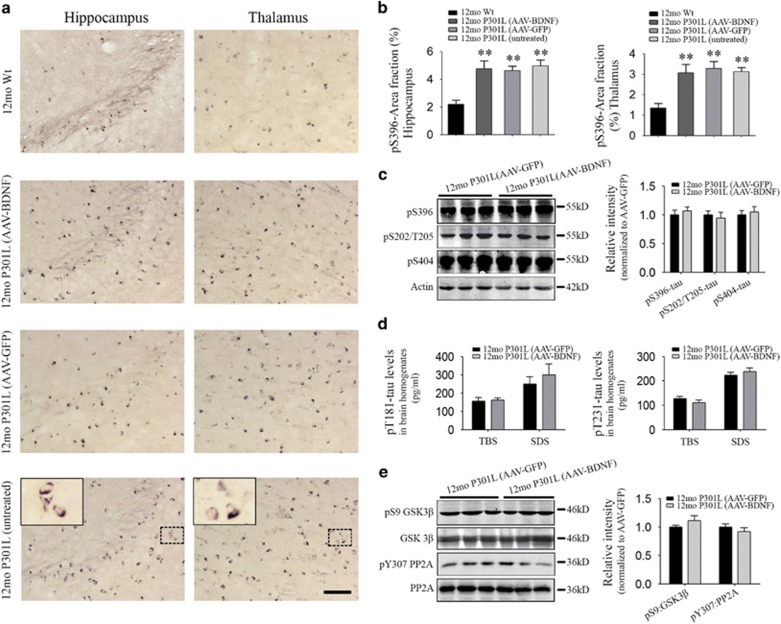
Genetic delivery of human brain-derived neurotrophic factor (BDNF) does not affect tau hyperphosphorylation. (**a**) Representative images of human pS396-tau immunostaining in the hippocampus and thalamus of different groups. Scale bar=100 μm. Insets show the representative morphology of pS396-tau-positive cells at higher magnification. (**b**) Comparison of the area fraction of human pS396-tau-positive immunostaining in the hippocampus (right panel) and thalamus (left panel) among the four groups (*n=*6 per group; mean±s.e.m., one-way analysis of variance (ANOVA), Tukey's test, **P<0.01). No differences were found among 12mo P301L (untreated), 12mo P301L (AAV-green fluorescent protein, AAV-GFP) and 12mo P301L (AAV-BDNF, adeno-associated virus carrying BDNF). (**c**) Representative western blot images and quantitative analyses of tau hyperphosphorylation levels for multiple sites in 12mo P301L (AAV-GFP) and 12mo P301L (AAV-BDNF) groups, including pS396, pS202/T025 and pS404 (*n=*6 per group, mean±s.e.m., Student's *t*-test, no difference was found between the two groups). (**d**) Quantification of human pT181 (right panel) and human pT231 (left panel) levels in Tris buffer solution (TBS) and sodium dodecyl sulfonate (SDS) extracts of P301L brain tissue homogenates from AAV-GFP and AAV-BDNF groups (*n=*6 per group, mean±s.e.m., Student's *t*-test, no difference was found between two groups). (**e**) Representative western blot images and quantitative analyses of the Tau phosphorylation kinase GSK3beta and the Tau phosphatase PP2A in brain tissue homogenates of P301L mice from two groups (*n=*6 per group, mean±s.e.m., Student's *t*-test, no difference was found between two groups). AAV, adeno-associated virus.

In order to investigate whether treatment of AAV-BDNF is able to change the levels of hyperphosphorylated tau, we compared the phosphorylated tau levels between AAV-BDNF and AAV-GFP groups by different regions, different tau aggregation states and different phosphorylation sites. In the hippocampus and thalamus, there were no differences in the area fraction and intensity of pS396-tau-positive staining among AAV-BDNF, AAV-GFP and untreated groups (Figure 6a, b). Using western blot assays, we also found no differences in multiple phosphorylation sites of tau between the AAV-GFP and AAV-BDNF treatments (Figure 6c). Tau was successively extracted in TBS and SDS, representing soluble and insoluble tau, respectively. In both TBS and SDS extracts, we found no differences in pT181- or pT231-tau levels between AAV-GFP and AAV-BDNF groups (Figure 6d). In addition, our data revealed that BDNF supplement did not affect the activities of tau phosphorylation enzyme GSK3β and phosphatase PP2A, as reflected by comparable levels of pS9-GSK3β:GSK3β and pY307-PP2A:PP2A in AAV-BDNF group to those in the AAV-GFP group (Figure 6e).

### Long-term expression of AAV-GFP or AAV-BDNF are well tolerated by mice

Throughout the animal studies, no obvious abnormal behavior was observed and no animal death occurred. Decreased levels of pro-inflammatory cytokines including interleukin (IL)-1β, tumor necrosis factor-α, interferon-γ and IL-6 was found in the brains of AAV-BDNF-treated P301L mice (Supplementary Figure 6a), indicating that delivery of AAV-BDNF did not induce damaging inflammatory reaction in brains, and inversely ameliorated neuroinflammation. Gene delivery did not significantly influence wet brain weight and animal body weight (Supplementary Figure 6b, c), nor liver enzyme activities (Supplementary Figure 6d, e). No discernible neoplasm was found in all AAV-administered animals. In addition, no sign of pathological morphologies was observed in organs including the intestine, kidney, liver, lung and heart (Supplementary Figure 6f). These data therefore suggest that long-term expression of recombinant human BDNF gene in the brain is well tolerated by P301L mice, demonstrating the safety of AAV-BDNF treatment.

## Discussion

In the present study, we confirmed BDNF deficiency in the serum and brain of AD patients and P301L mice. We further found that AAV-BDNF gene delivery reduced behavioral deficits, prevented neuron loss, alleviated synaptic degeneration and attenuated neurogenesis impairment in P301L mice. Although BDNF supplement seemed not to affect tau hyperphosphorylation levels, the treatment with BDNF presents itself as a promising treatment for tau-related neurodegeneration because of its neuroprotective effect and the advantage of its safety and the long-term expression.

A great challenge in the field of neurotrophin therapy is drug delivery to the central nervous system. When administered via peripheral administration, only limited amounts of BDNF can cross the blood–brain barrier owing to its charge and molecular size.^16^ The AAV delivery strategy may address these issues. First, therapeutic proteins encoded by recombinant AAVs are widely and stably expressed, allowing its actions on target neurons in the brain regions of interest, and allowing its long-term treatment. Second, to date, AAV treatment is considered relatively safe,^27, 28^ as evidenced by the Food and Drug Administration-approved clinical trials of AAV-based gene therapies. In the present study, we found that after AAV-BDNF delivery, BDNF was stably expressed until the end point of the study, and also widely expressed throughout different brain regions. Our data further demonstrate the safety of brain delivery of BDNF gene via AAV. In the present study, AAV-BDNF therapy is well tolerated in animals. Another neurotrophin, nerve growth factor, is used in humans for AD treatment but leads to pain.^29^ In this regard, BDNF seems to be more appropriate for use in humans than nerve growth factor. The safety and effectiveness of BDNF for AD treatment needs to be validated in the future.

Accumulating evidence reveals that there are two forms of BDNF: pro-BDNF and mature BDNF. Pro-BDNF is a high-affinity ligand for neurotrophin receptor p75 (p75NTR) and sortilin,^30^ whereas mature BDNF binds with a greatest affinity to tropomyosin-related kinase receptor type B.^31^ The distinction into BDNF isoform binding determines their differential physiological functions, as the activation of the pro-BDNF/p75NTR/sortilin pathway results in apoptosis^32^ and neurite collapse,^33^ and conversely, the activation of the BDNF/tropomyosin-related kinase receptor type B pathway predominantly supports neuronal survival.^34^ In light of what was discussed above, we used AAV-mediated gene transfer to ensure a beneficial outcome of the AAV-BDNF application. As expected, we indeed observed these beneficial effects, including improvement of behavioral performances, attenuation of neuronal loss, and prevention for synaptic degeneration and neurogenesis impairment.

Of note, we found no effect of BDNF supplementation on tau hyperphosphorylation, indicating that BDNF may have no direct or indirect action on the tau protein, but rather interfere with tau's downstream toxicity. This result is consistent with previous findings that the genetic knockdown of BDNF in 3 × Tg-AD mice with mutant human presenilin-1, APP and human tau did not alter tau phosphorylation levels.^35^ In contrast, in an *in vitro* model of differentiated neurons from embryonic carcinoma cells, BDNF was found to cause a rapid decline of phosphorylated tau levels,^36^ and in rat cerebellar granule cells, similarly, BDNF induces a rapid upregulation of dephosphorylated tau.^37^ This contradiction may be because of differences in the models used (*in vivo* versus *in vitro*). It is likely that a rapid downregulation of phosphorylated tau appears as an acute response of neurons to BDNF supplementation *in vitro*. However, after long-term BDNF treatment *in vivo*, tau phosphorylation and dephosphorylation may tend to become balanced because of complicated physiological processes, in particular the co-regulation of tau phosphorylation kinase GSK3β and phosphatase PP2A.^38^ Indeed, we did not find any significant changes in the activities of these two enzymes in the present study. Thus, we speculated that BDNF may have no 'direct' action on tau protein, but rather exert 'supportive effects' to operate downstream of tau hyperphosphorylation. The mechanisms underlying these 'supportive effects' are mainly because of the activation of the BDNF/tropomyosin-related kinase receptor type B signaling pathway, involving the downstreaming signaling of the mitogen-activated protein kinase kinase (MEK)–extracellular signal-regulated kinase (ERK) pathway, the phosphorylation of phosphoinositide 3-kinase (PI3K) pathway and phospholipase Cγ1 pathway.^16^ Both MEK–ERK and PI3K pathways are deemed implicated in neurite outgrowth and neuronal survival, and the phospholipase Cγ1 pathway predominantly in synaptic plasticity via the mobilization of calcium stores and the activation of calcium-dependent protein kinases.^34^

On the basis of our findings, we propose here that BDNF may exert a potent 'supportive effect' on neuron and synapse by antagonizing harmful pathological events derived from tau hyperphosphorylation. In light of this point, the delivery of AAV-BDNF may be seen as a support therapy to tauopathy. Tauopathy is an integral phenotype of AD, as well as several related disorders including frontotemporal dementia, Pick's disease, Huntington's disease, progressive supranuclear palsy, corticobasal degeneration, argyrophilic grain disease, tangle-only dementia, white matter tauopathy with globular glial inclusions and Down's syndrome.^39, 40, 41, 42^ Recent studies showed that reduced BDNF expression and BDNF polymorphisms also existed in other diseases with tauopathies. In patients with Pick' disease, the Val66Met genotype occurred more frequently,^43^ and the BDNF mRNA was downregulated in the parietal cortex.^44^ Reduced protein and mRNA levels of BDNF have also been found in brain tissue derived from mouse models of Huntington's disease,^45^ and in the caudate and putamen of patients with Huntington's disease.^46^ Furthermore, BDNF may have a role in the pathogenesis of corticobasal degeneration because BDNF mRNA levels were reduced in the parietal cortex of patients with this disease.^44^ In our study, the BDNF levels were reduced in the brain of P301L mouse, which is also a model for frontotemporal dementia, whereas preserved BDNF expression has been observed in the cerebral cortex of patients suffering from frontotemporal dementia.^47^ Further studies are needed to confirm BDNF expression and signal pathway in patients and animal models of other disorders with tauopathies, especially frontotemporal dementia. In spite of this, given that BDNF polymorphism and reduction are related with AD and other diseases with tauopathies, and the protective effect of BDNF in P301L mice, BDNF treatment might also be beneficial in other neurodegenerative diseases with tauopathies.

In conclusion, the present study uncovered the therapeutic effects of AAV-BDNF on tauopathy, suggesting that BDNF supplement is a promising strategy for prevention and treatment of AD and other neurodegenerative diseases with tauopathies.

## References

[bib1] Arriagada PV, Growdon JH, Hedley-Whyte ET, Hyman BT. Neurofibrillary tangles but not senile plaques parallel duration and severity of Alzheimer's disease. Neurology 1992; 42: 631. 1549228 10.1212/wnl.42.3.631

[bib2] Gomez-Isla T, Hollister R, West H, Mui S, Growdon JH, Petersen RC et al. Neuronal loss correlates with but exceeds neurofibrillary tangles in Alzheimer's disease. Ann Neurol 1997; 41: 17–24. 9005861 10.1002/ana.410410106

[bib3] Bateman RJ, Xiong C, Benzinger TL, Fagan AM, Goate A, Fox NC et al. Clinical and biomarker changes in dominantly inherited Alzheimer's disease. N Engl J Med 2012; 367: 795–804. 22784036 10.1056/NEJMoa1202753 PMC3474597

[bib4] Ittner LM, Ke YD, Delerue F, Bi M, Gladbach A, van Eersel J et al. Dendritic function of tau mediates amyloid-beta toxicity in Alzheimer's disease mouse models. Cell 2010; 142: 387–397. 20655099 10.1016/j.cell.2010.06.036

[bib5] Roberson ED, Scearce-Levie K, Palop JJ, Yan F, Cheng IH, Wu T et al. Reducing endogenous tau ameliorates amyloid beta-induced deficits in an Alzheimer's disease mouse model. Science 2007; 316: 750–754. 17478722 10.1126/science.1141736

[bib6] Diniz BS, Teixeira AL. Brain-derived neurotrophic factor and Alzheimer's disease: physiopathology and beyond. Neuromol Med 2011; 13: 217–222. 10.1007/s12017-011-8154-x 21898045

[bib7] Kunugi H, Ueki A, Otsuka M, Isse K, Hirasawa H, Kato N et al. A novel polymorphism of the brain-derived neurotrophic factor (BDNF) gene associated with late-onset Alzheimer's disease. Mol Psychiatry 2001; 6: 83–86. 11244490 10.1038/sj.mp.4000792

[bib8] Tsai SJ, Hong CJ, Liu HC, Liu TY, Hsu LE, Lin CH. Association analysis of brain-derived neurotrophic factor Val66Met polymorphisms with Alzheimer's disease and age of onset. Neuropsychobiology 2004; 49: 10–12. 14730194 10.1159/000075332

[bib9] Tsai SJ, Hong CJ, Liu HC, Liu TY, Liou YJ. The brain-derived neurotrophic factor gene as a possible susceptibility candidate for Alzheimer's disease in a chinese population. Dement Geriatr Cogn Disord 2006; 21: 139–143. 16391475 10.1159/000090673

[bib10] Fukumoto N, Fujii T, Combarros O, Kamboh MI, Tsai SJ, Matsushita S et al. Sexually dimorphic effect of the Val66Met polymorphism of BDNF on susceptibility to Alzheimer's disease: New data and meta-analysis. Am J Med Genet B 2010; 153B: 235–242. 10.1002/ajmg.b.30986 19504537

[bib11] Laske C, Stransky E, Leyhe T, Eschweiler GW, Maetzler W, Wittorf A et al. BDNF serum and CSF concentrations in Alzheimer's disease, normal pressure hydrocephalus and healthy controls. J Psychiatr Res 2007; 41: 387–394. 16554070 10.1016/j.jpsychires.2006.01.014

[bib12] Lee JG, Shin BS, You YS, Kim JE, Yoon SW, Jeon DW et al. Decreased serum brain-derived neurotrophic factor levels in elderly korean with dementia. Psychiatry Investig 2009; 6: 299–305. 10.4306/pi.2009.6.4.299 PMC2808800 20140129

[bib13] Holsinger RM, Schnarr J, Henry P, Castelo VT, Fahnestock M. Quantitation of BDNF mRNA in human parietal cortex by competitive reverse transcription-polymerase chain reaction: decreased levels in Alzheimer's disease. Brain Res Mol Brain Res 2000; 76: 347–354. 10762711 10.1016/s0169-328x(00)00023-1

[bib14] Beeri MS, Sonnen J. Brain BDNF expression as a biomarker for cognitive reserve against Alzheimer disease progression. Neurology 2016; 86: 702–703. 26819454 10.1212/WNL.0000000000002389

[bib15] Buchman AS, Yu L, Boyle PA, Schneider JA, De Jager PL, Bennett DA. Higher brain BDNF gene expression is associated with slower cognitive decline in older adults. Neurology 2016; 86: 735–741. 26819457 10.1212/WNL.0000000000002387 PMC4763800

[bib16] Nagahara AH, Tuszynski MH. Potential therapeutic uses of BDNF in neurological and psychiatric disorders. Nat Rev Drug Discov 2011; 10: 209–219. 21358740 10.1038/nrd3366

[bib17] Arancibia S, Silhol M, Mouliere F, Meffre J, Hollinger I, Maurice T et al. Protective effect of BDNF against beta-amyloid induced neurotoxicity *in vitro* and *in vivo* in rats. Neurobiol Dis 2008; 31: 316–326. 18585459 10.1016/j.nbd.2008.05.012

[bib18] Zhang L, Fang Y, Lian Y, Chen Y, Wu T, Zheng Y et al. Brain-derived neurotrophic factor ameliorates learning deficits in a rat model of Alzheimer's disease induced by abeta1-42. PLoS One 2015; 10: e0122415. 25849905 10.1371/journal.pone.0122415 PMC4388634

[bib19] Nagahara AH, Merrill DA, Coppola G, Tsukada S, Schroeder BE, Shaked GM et al. Neuroprotective effects of brain-derived neurotrophic factor in rodent and primate models of Alzheimer's disease. Nat Med 2009; 15: 331–337. 19198615 10.1038/nm.1912 PMC2838375

[bib20] Nagahara AH, Mateling M, Kovacs I, Wang L, Eggert S, Rockenstein E et al. Early BDNF treatment ameliorates cell loss in the entorhinal cortex of APP transgenic mice. J Neurosci 2013; 33: 15596–15602. 24068826 10.1523/JNEUROSCI.5195-12.2013 PMC3782628

[bib21] Deters N, Ittner LM, Gotz J. Divergent phosphorylation pattern of tau in P301L tau transgenic mice. Eur J Neurosci 2008; 28: 137–147. 18662339 10.1111/j.1460-9568.2008.06318.x

[bib22] Ramsden M, Kotilinek L, Forster C, Paulson J, McGowan E, SantaCruz K et al. Age-dependent neurofibrillary tangle formation, neuron loss, and memory impairment in a mouse model of human tauopathy (P301L). J Neurosci 2005; 25: 10637–10647. 16291936 10.1523/JNEUROSCI.3279-05.2005 PMC6725849

[bib23] Xiang Y, Bu XL, Liu YH, Zhu C, Shen LL, Jiao SS et al. Physiological amyloid-beta clearance in the periphery and its therapeutic potential for Alzheimer's disease. Acta Neuropathol 2015; 130: 487–499. 26363791 10.1007/s00401-015-1477-1 PMC4575389

[bib24] Imayoshi I, Sakamoto M, Ohtsuka T, Takao K, Miyakawa T, Yamaguchi M et al. Roles of continuous neurogenesis in the structural and functional integrity of the adult forebrain. Nat Neurosci 2008; 11: 1153–1161. 18758458 10.1038/nn.2185

[bib25] Westerlund N, Zdrojewska J, Padzik A, Komulainen E, Bjorkblom B, Rannikko E et al. Phosphorylation of SCG10/stathmin-2 determines multipolar stage exit and neuronal migration rate. Nat Neurosci 2011; 14: 305–313. 21297631 10.1038/nn.2755

[bib26] Santacruz K, Lewis J, Spires T, Paulson J, Kotilinek L, Ingelsson M et al. Tau suppression in a neurodegenerative mouse model improves memory function. Science 2005; 309: 476–481. 16020737 10.1126/science.1113694 PMC1574647

[bib27] Kaspar BK, Vissel B, Bengoechea T, Crone S, Randolph-Moore L, Muller R et al. Adeno-associated virus effectively mediates conditional gene modification in the brain. Proc Natl Acad Sci USA 2002; 99: 2320–2325. 11842206 10.1073/pnas.042678699 PMC122363

[bib28] Shen F, Kuo R, Milon-Camus M, Han Z, Jiang L, Young WL et al. Intravenous delivery of adeno-associated viral vector serotype 9 mediates effective gene expression in ischemic stroke lesion and brain angiogenic foci. Stroke 2013; 44: 252–254. 23250995 10.1161/STROKEAHA.112.662965 PMC3531817

[bib29] Eriksdotter Jonhagen M, Nordberg A, Amberla K, Backman L, Ebendal T, Meyerson B et al. Intracerebroventricular infusion of nerve growth factor in three patients with Alzheimer's disease. Dement Geriatr Cogn Disord 1998; 9: 246–257. 9701676 10.1159/000017069

[bib30] Teng HK, Teng KK, Lee R, Wright S, Tevar S, Almeida RD et al. ProBDNF induces neuronal apoptosis via activation of a receptor complex of p75NTR and sortilin. J Neurosci 2005; 25: 5455–5463. 15930396 10.1523/JNEUROSCI.5123-04.2005 PMC6724992

[bib31] Lee R, Kermani P, Teng KK, Hempstead BL. Regulation of cell survival by secreted proneurotrophins. Science 2001; 294: 1945–1948. 11729324 10.1126/science.1065057

[bib32] Koshimizu H, Hazama S, Hara T, Ogura A, Kojima M. Distinct signaling pathways of precursor BDNF and mature BDNF in cultured cerebellar granule neurons. Neurosci Lett 2010; 473: 229–232. 20219632 10.1016/j.neulet.2010.02.055

[bib33] Sun Y, Lim Y, Li F, Liu S, Lu JJ, Haberberger R et al. ProBDNF collapses neurite outgrowth of primary neurons by activating RhoA. PLoS one 2012; 7: e35883. 22558255 10.1371/journal.pone.0035883 PMC3338794

[bib34] Kaplan DR, Miller FD. Neurotrophin signal transduction in the nervous system. Curr Opin Neurobiol 2000; 10: 381–391. 10851172 10.1016/s0959-4388(00)00092-1

[bib35] Castello NA, Green KN, LaFerla FM. Genetic knockdown of brain-derived neurotrophic factor in 3xTg-AD mice does not alter Abeta or tau pathology. PLoS One 2012; 7: e39566. 22870188 10.1371/journal.pone.0039566 PMC3411687

[bib36] Elliott E, Atlas R, Lange A, Ginzburg I. Brain-derived neurotrophic factor induces a rapid dephosphorylation of tau protein through a PI-3 Kinase signalling mechanism. Eur J Neurosci 2005; 22: 1081–1089. 16176349 10.1111/j.1460-9568.2005.04290.x

[bib37] Coffey ET, Akerman KE, Courtney MJ. Brain derived neurotrophic factor induces a rapid upregulation of synaptophysin and tau proteins via the neurotrophin receptor TrkB in rat cerebellar granule cells. Neurosci Lett 1997; 227: 177–180. 9185679 10.1016/s0304-3940(97)00335-2

[bib38] Gotz J, Gladbach A, Pennanen L, van Eersel J, Schild A, David D et al. Animal models reveal role for tau phosphorylation in human disease. Biochim Biophys Acta 2010; 1802: 860–871. 19751831 10.1016/j.bbadis.2009.09.008

[bib39] Goedert M, Clavaguera F, Tolnay M. The propagation of prion-like protein inclusions in neurodegenerative diseases. Trends Neurosci 2010; 33: 317–325. 20493564 10.1016/j.tins.2010.04.003

[bib40] Buee L, Bussiere T, Buee-Scherrer V, Delacourte A, Hof PR. Tau protein isoforms, phosphorylation and role in neurodegenerative disorders. Brain Res Brain Res Rev 2000; 33: 95–130. 10967355 10.1016/s0165-0173(00)00019-9

[bib41] Fernandez-Nogales M, Cabrera JR, Santos-Galindo M, Hoozemans JJ, Ferrer I, Rozemuller AJ et al. Huntington's disease is a four-repeat tauopathy with tau nuclear rods. Nat Med 2014; 20: 881–885. 25038828 10.1038/nm.3617

[bib42] Irwin DJ, Lee VM, Trojanowski JQ. Parkinson's disease dementia: convergence of alpha-synuclein, tau and amyloid-beta pathologies. Nat Rev Neurosci 2013; 14: 626–636. 23900411 10.1038/nrn3549 PMC4017235

[bib43] Feher A, Juhasz A, Rimanoczy A, Kalman J, Janka Z. Association between BDNF Val66Met polymorphism and Alzheimer disease, dementia with Lewy bodies, and Pick disease. Alzheimer DIs Assoc Disord 2009; 23: 224–228. 19812463 10.1097/WAD.0b013e318199dd7d

[bib44] Belrose JC, Masoudi R, Michalski B, Fahnestock M. Increased pro-nerve growth factor and decreased brain-derived neurotrophic factor in non-Alzheimer's disease tauopathies. Neurobiol Aging 2014; 35: 926–933. 24112788 10.1016/j.neurobiolaging.2013.08.029

[bib45] Zuccato C, Cattaneo E. Brain-derived neurotrophic factor in neurodegenerative diseases. Nat Rev Neurol 2009; 5: 311–322. 19498435 10.1038/nrneurol.2009.54

[bib46] Ferrer I, Goutan E, Marin C, Rey MJ, Ribalta T. Brain-derived neurotrophic factor in Huntington disease. Brain Res 2000; 866: 257–261. 10825501 10.1016/s0006-8993(00)02237-x

[bib47] Ferrer I, Marin C, Rey MJ, Ribalta T. Brain-derived neurotrophic factor in patients with frontotemporal dementia. Neurosci Lett 2000; 279: 33–36. 10670781 10.1016/s0304-3940(99)00937-4

