# Supplementary material for: Brain-derived neurotrophic factor protects against tau-related neurodegeneration of Alzheimer's disease
Source: Transl Psychiatry. 2016 Oct 4;6(10):e907–. doi: 10.1038/tp.2016.186 (PMC5315549; doi:10.1038/tp.2016.186)
Supplement: Supplementary Figures and Table [file tp2016186x1.doc]

**Supplementary Figure 1**

**
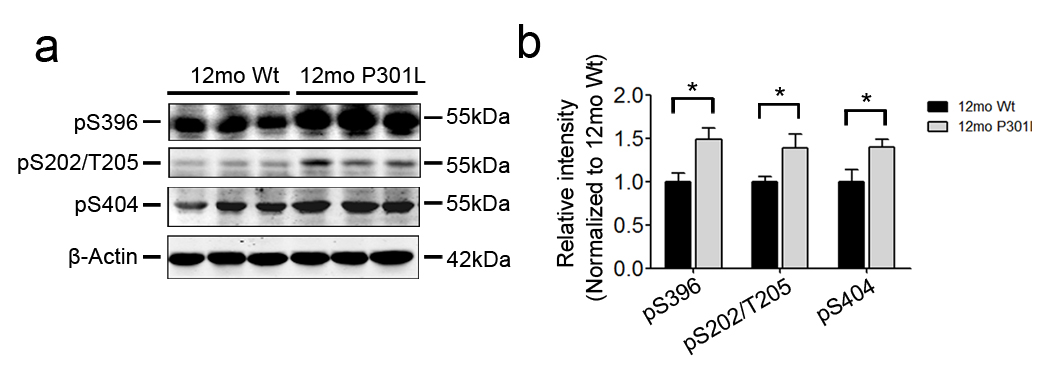
**

**Supplementary Figure 1. At 12 months of age, P301L mice have higher phosphorylated tau levels than their wild-type littermates. (a)** and **(b)**, Representative Western blot images (**a**) and quantification (**b**) of phosphorylated tau at multiple sites including S396, S202/T205 and S404 in brain homogenates (n = 6 for each group, mean ± s.e.m., Student's t-test, **P*< 0.05). 12mo P301L denotes 12-month old P301L transgenic mice; 12mo Wt denotes 12-month old wild type littermates.

**Supplementary Figure 2**

**
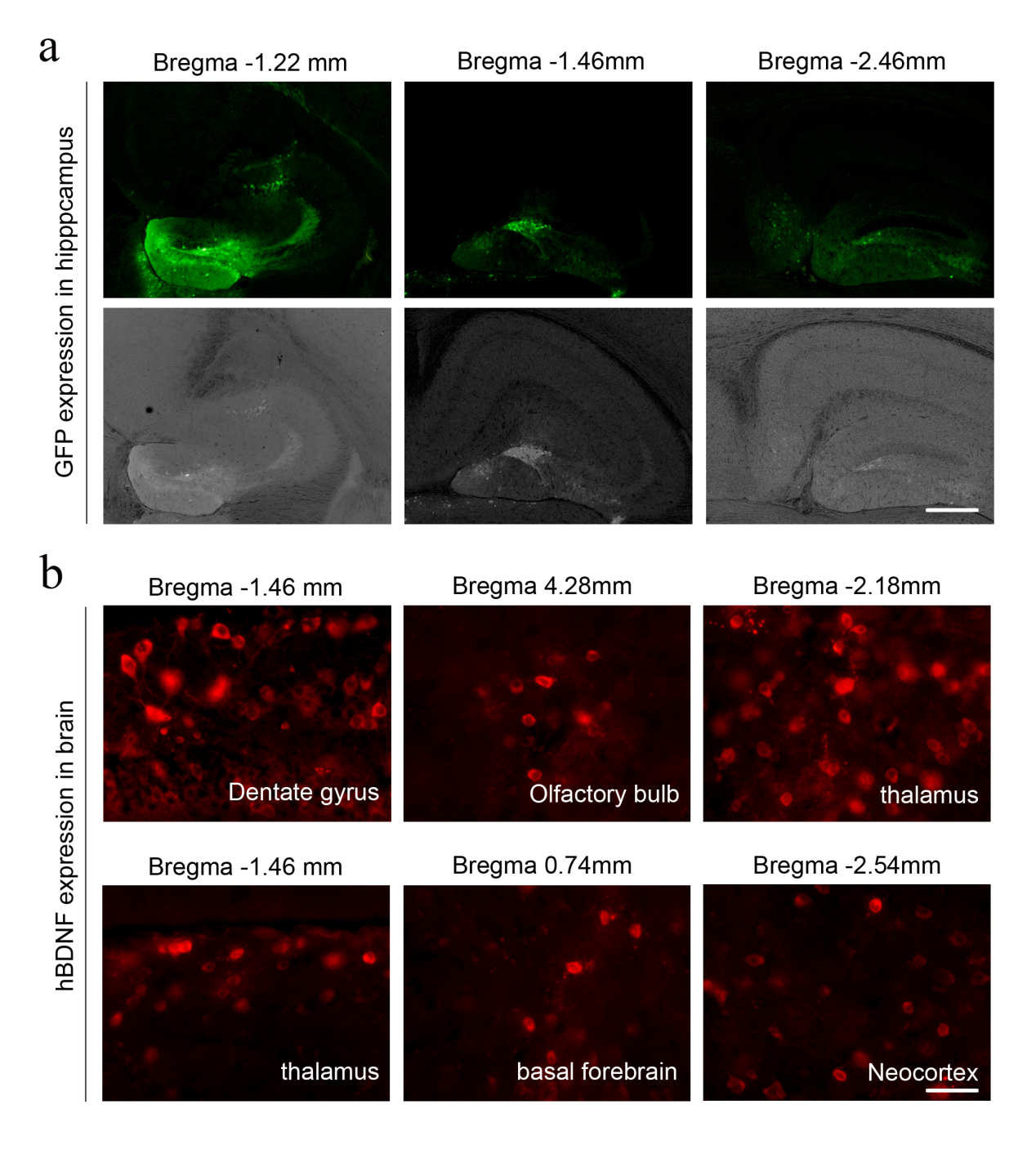
**

**Supplementary Figure 2. (a)**, GFP expression was detected throughout multiple subregions of the hippocampus in P301L mice at 9 months after AAV-GFP injection. Scale bar=400μm. **(b)**, Distribution of human mature BDNF (hBDNF) expression in brain sagittal sections of 12-month old P301L mice at 9 months after AAV-BDNF injection Scale bar=25μm.

**Supplementary Figure 3**

­
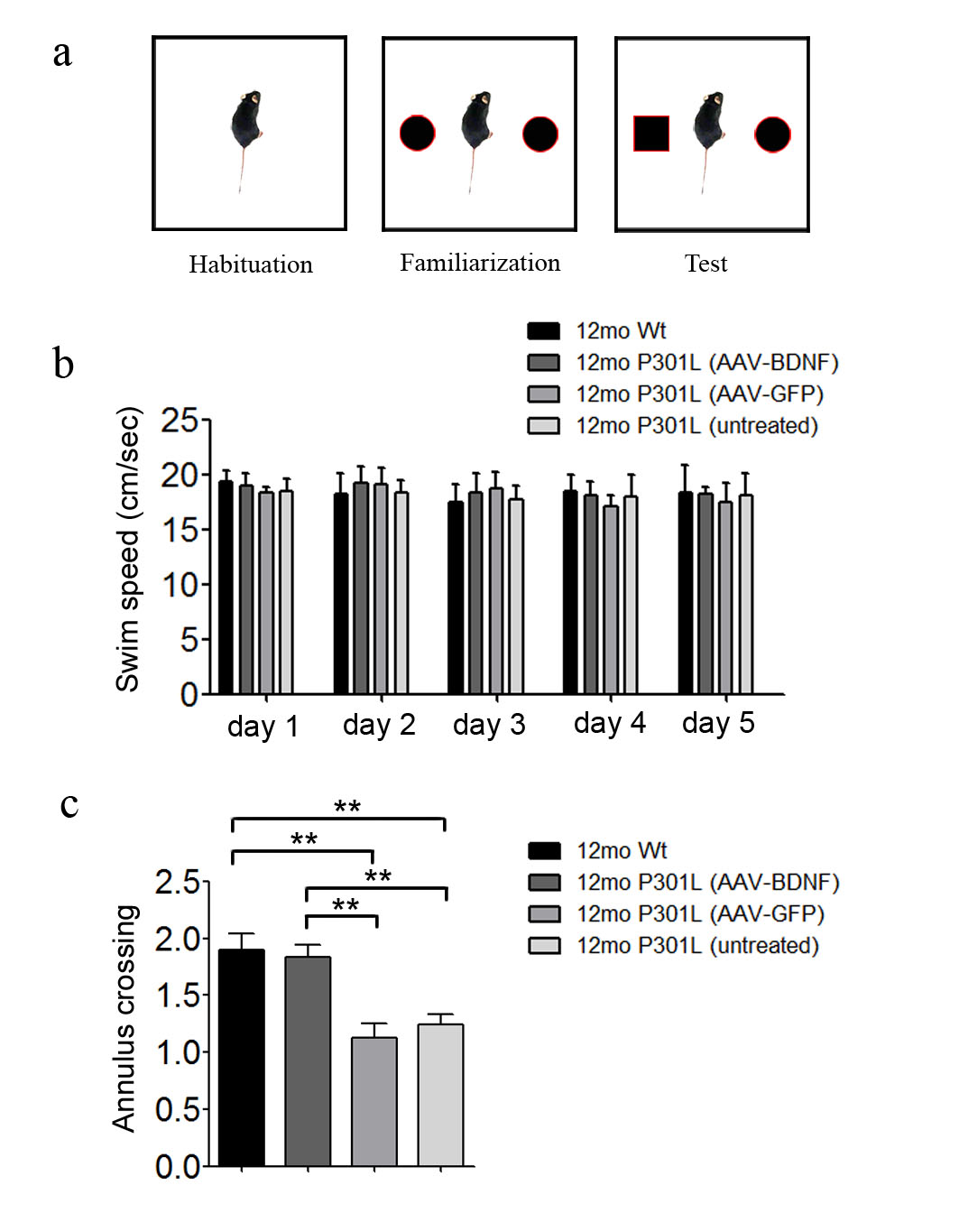


**Supplementary Figure 3. (a)**, Experimental design of the novel object recognition test. **(b)**, Swim speed (cm/sec) calculated by the equation, velocity = distance/time, during platform trials in Morris water maze were analyzed (n=8 per group, mean ± s.e.m., two way ANOVA, Tukey’s test). There was no difference in swim speed among groups. **(c)**, Comparison of number of annulus crossing in probe test among groups (n=8 per group, mean ± s.e.m., one way ANOVA, Tukey’s test, ***P*<0.01).

**Supplementary Figure 4**

**
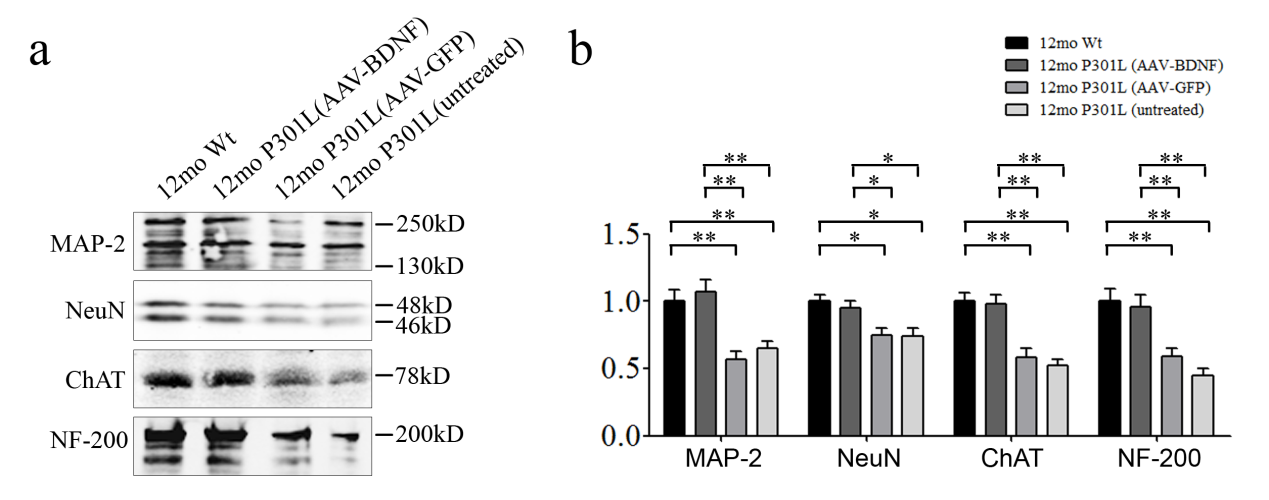
Supplementary Figure 4.** The expression of MAP-2, NeuN, ChAT and NF-200 in brain homogenates of 12mo Wt, 12mo P301L (AAV-BDNF), 12mo P301L (AAV-GFP) and 12mo P301L (untreated) mice, assessed by Western blot analysis (n=6 per group; mean ± s.e.m., one-way ANOVA, Tukey’s test, **P*<0.05, ***P*<0.01).

**Supplementary Figure 5**

**
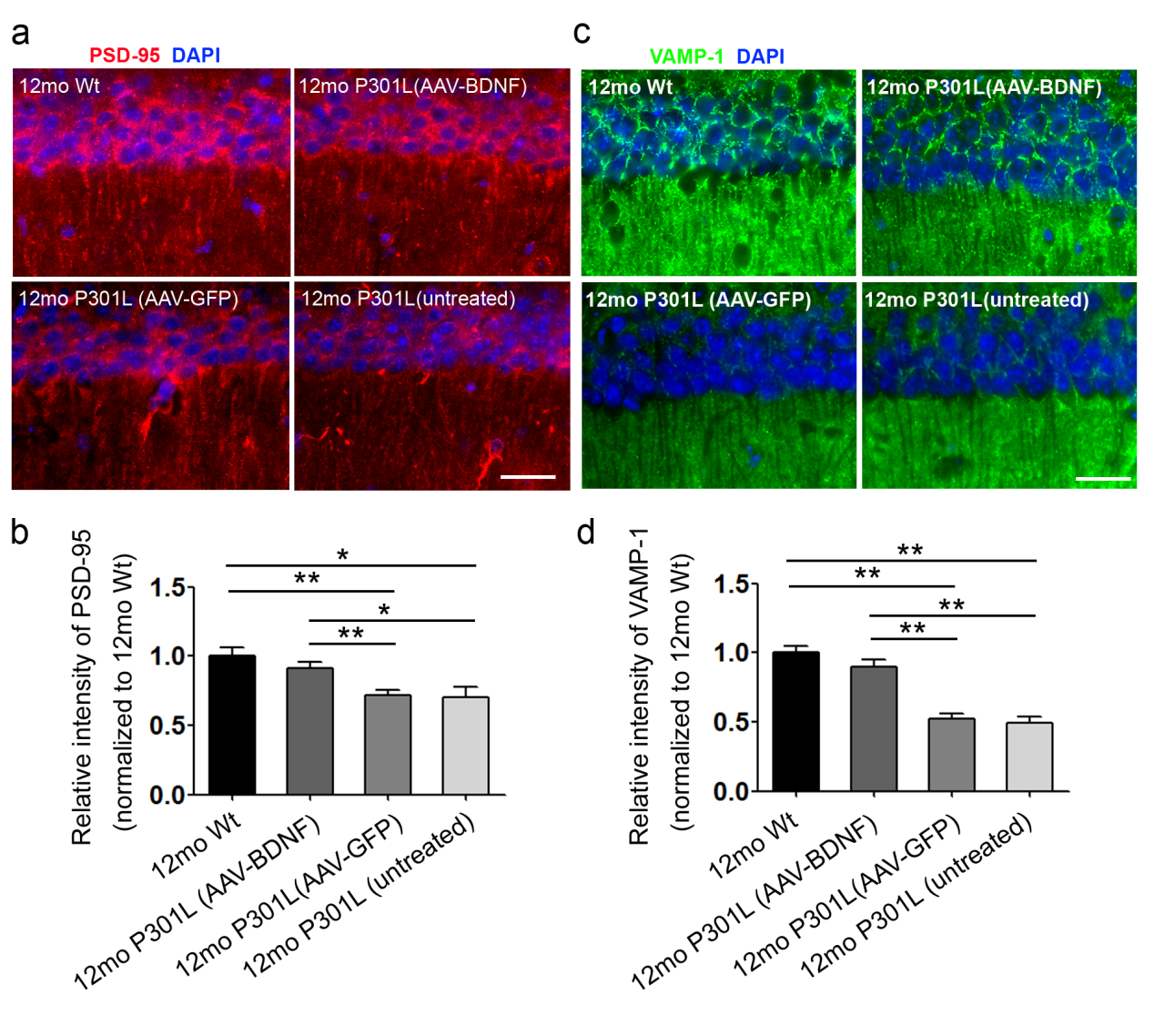
**

**Supplementary Figure 5. (a)**, The expression of PSD-95 in the hippocampus of 12mo Wt, 12mo P301L (AAV-BDNF), 12mo P301L (AAV-GFP) and 12mo P301L (untreated) mice, assessed by PSD-95 and DAPI double immunofluorescence (Scale bar=50μm). **(b)**, Quantification of PSD-95 expression in the hippocampus (n=6 per group; mean ± s.e.m., one-way ANOVA, Tukey’s test, **P*<0.05, ***P*<0.01). **(c)** and **(d)**, Representative images (**c**) and quantification (**d**) of VAMP-1 expression in the hippocampus (n=6 per group; mean ± s.e.m., one-way ANOVA, Tukey’s test, ***P*<0.01. Scale bar=50μm).

**Supplementary Figure 6**

**
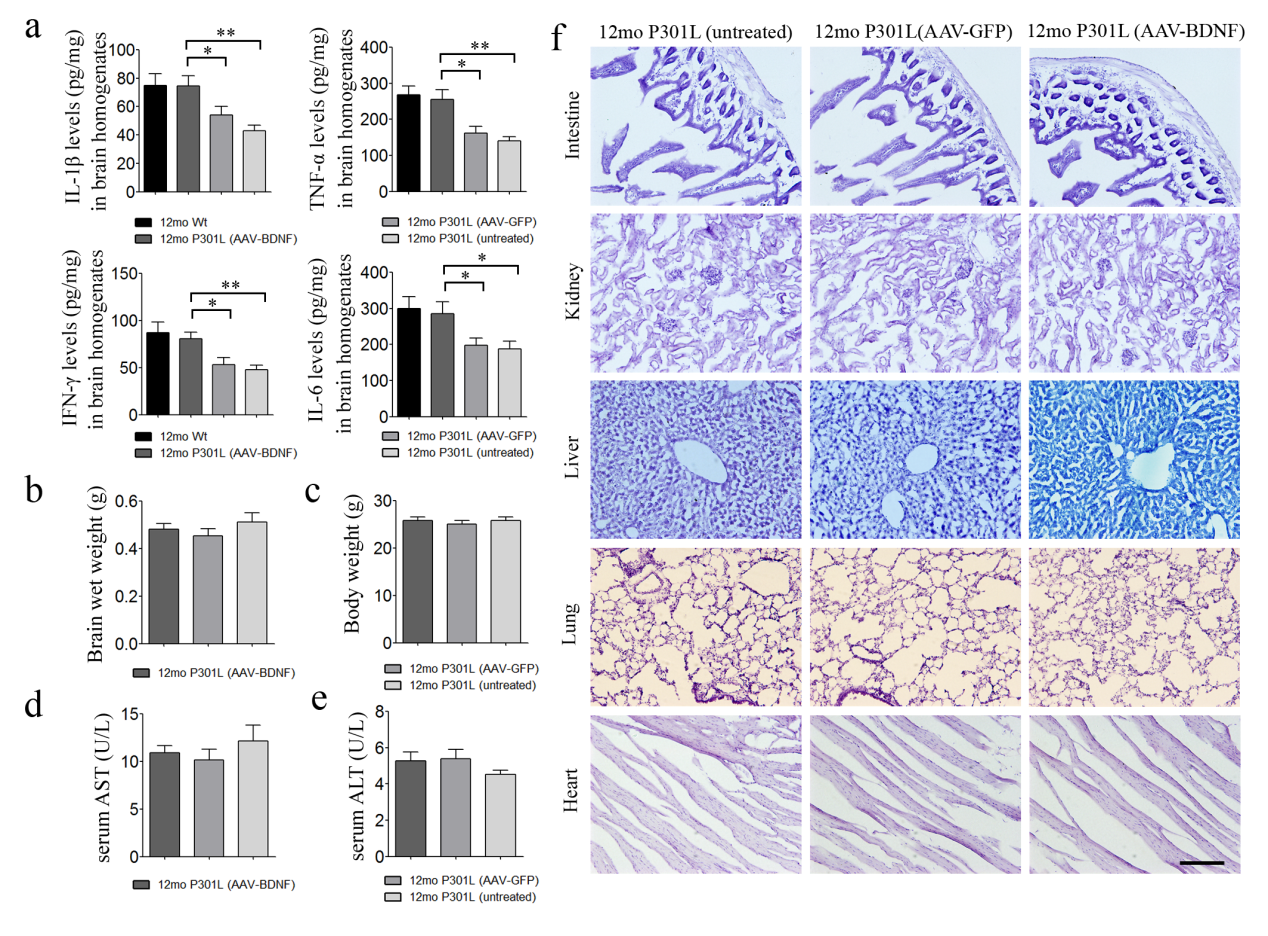
**

**Supplementary Figure 6. Genetic delivery of human BDNF is well tolerated. (a)**, Levels of IL-1β, TNF-α, IFN-γ and IL-6 in brain homogenates of 12-month-old P30L mice (untreated, AAV-GFP treated, AAV-BDNF treated) and Wt littermates (n=6 per group, mean ± s.e.m., one-way ANOVA, Tukey’s test, **P*<0.05, ***P*<0.01). **(b)**-**(e)**, No differences in wet brain weight (**b**), body weight (**c)**, AST (aspartic transaminase) (**d**) and ALT (alanine transaminase) (**e**) among 12-month-old P301L mice with different treatments (n=6 per group, mean ± s.e.m., one-way ANOVA, Tukey’s test). **(f)**, Hematoxylin-eosin staining shows no discernible pathological morphology in the intestine, kidney, liver, lung and heart after 9 months of AAV injection (n = 6 for each group). Scale bar= 200 μm.

**Supplementary Table. Demographic and clinical characteristics of patients with Alzheimer’s Disease and healthy elderly controls.**

| Variables | AD, n=44 | HE, n=54 | *p* value |
| --- | --- | --- | --- |
| Age, y, mean±SD | 72.48±8.22 | 70.59±6.33 | 0.203 |
| Female, n(%) | 27(61.36) | 33(61.11) | 0.980 |
| Education, y, mean±SD | 7.64±4.36 | 9.98±3.70 | 0.005 |
| MMSE score, mean±SD | 12.45±5.93 | 28.48±2.03 | <0.001 |
| CDR score, mean±SD | 1.58±0.82 | 0 | <0.001 |

AD, Alzheimer’s disease; HE, healthy elderly controls; MMSE, mini-mental state examination; CDR, clinical dementia rating.
